# Cytological diploidization of paleopolyploid genus *Zea*: Divergence between homoeologous chromosomes or activity of pairing regulator genes?

**DOI:** 10.1371/journal.pone.0189644

**Published:** 2018-01-02

**Authors:** Lidia Poggio, Graciela Esther González

**Affiliations:** Instituto de Ecología, Genética y Evolución (IEGEBA, Consejo Nacional de Investigaciones Científicas y Técnicas—CONICET)—Laboratorio de Citogenética y Evolución (LaCyE), Departamento de Ecología, Genética y Evolución, Facultad de Ciencias Exactas y Naturales, Universidad de Buenos Aires, Ciudad Autónoma de Buenos Aires, Argentina; The Institute of Genetics and Developmental Biology (IGDB) of the Chinese Academy of Sciences (CAS), China, CHINA

## Abstract

Cytological diploidization process is different in autopolyploid and allopolyploid species. Colchicine applied at the onset of meiosis suppresses the effect of pairing regulator genes resulting multivalents formation in bivalent-forming species. Colchicine treated maizes (4x = 2n = 20, A_m_A_m_B_m_B_m_) showed up to 5IV, suggesting pairing between chromosomes from genomes homoeologous A_m_ and B_m_. In untreated individuals of the alloautooctoploid *Zea perennis* (8x = 2n = 40, A_p_A_p_A_p´_A_p´_B_p1_B_p1_B_p2_B_p2_) the most frequent configuration was 5IV+10II (formed by A and B genomes, respectively). The colchicine treated *Z*. *perennis* show up to 10IV revealing higher affinity within genomes A and B, but any homology among them. These results suggest the presence of a paring regulator locus (*PrZ*) in maize and *Z*. *perennis*, whose expression is suppressed by colchicine. It could be postulated that in *Z*. *perennis*, *PrZ* would affect independently the genomes A and B, being relevant the threshold of homology, the fidelity of pairing in each genomes and the ploidy level. Cytological analysis of the treated hexaploid hybrids (6x = 2n = 30), with *Z*. *perennis* as a parental, strongly suggests that *PrZ* is less effective in only one doses. This conclusion was reinforced by the homoeologous pairing observed in untreated dihaploid maizes, which showed up to 5II. Meiotic behaviour of individuals treated with different doses of colchicine allowed to postulate that *PrZ* affect the homoeologous association by controlling entire genomes (A_m_ or B_m_) rather than individual chromosomes. Based on cytological and statistical results it is possible to propose that the cytological diploidization in *Zea* species occurs by restriction of pairing between homoeologous chromosomes or by genetical divergence of the homoeologous chromosomes, as was observed in untreated *Z*. *mays* ssp. *parviglumis*. These are independent but complementary systems and could be acting jointly in the same nucleus.

## Introduction

Polyploidy, the presence of two or more genomes per cell, has played a major role in the evolution of higher plants. It has been clearly established that most of the extant flowering plants are polyploids or paleopolyploids species [1, 2]. The result of continued polyploidization in different anastomosing lineages is a polyploidy complex. In the course of time the diploid members or the lower polyploids of a series may become extinct from a genus or even a family, leaving the higher polyploids behind at the one living representatives of the complex (ancient polyploids or paleopolyploids). Polyploidy vary along a continuum from homogeneous (autopolyploids) to partially divergent (segmental allopolyploids) and highly divergent genomes (typical allopolyploids) [3].

Genetic determination of exclusive pairing between homologous chromosomes or “cytological diploidization” is the process by which meiosis in polyploids leads to chromosomally and genetically balanced gametes. The process of suppression of homoeologous pairing is the key to success of many polyploids species. The diploid-like meiotic behaviour of polyploids could be the result of the divergence between homoeologous chromosomes or by genetic control [1, 3–9].

Little is known about the activity of genes contributing to the cytological diploidization of polyploids and their dihaploids. The best understood is the *Ph1* locus of polyploid wheat, which suppresses pairing between homoeologous genomes. The best understood is the *Ph1* locus of polyploid wheat, which suppresses pairing between homoeologous chromosomes. There are evidences suggesting that this gene is involved in the onset of meiosis and have functions controlling the transcription of meiotic genes. It has also been reported that *Ph1* contributes to the clustering of the telomeres at nuclear membrane as a bouquet, facilitating homologue recognition, and is involved in the chromatin remodeling. There are evidences that this locus could contribute to the fidelity of synapses and crossover formation [6, 8–12]. *Ph1*-like genes were also postulated in other sexually propagating polyploids, such as *Avena sativa*, *Festuca arundinacea*, *Brassica napus*, *Gossypium hirsutum* and *Gossypium barbadense*, as well as in some diploids from genera *Lolium* and *Glandularia* [1, 9, 13].

Jenczewsky and colleagues [1] reviewed diploidization in polyploids from a cytological, genetic, agronomic and evolutionary point of view. All this approach pointed out that suppression of crossing over between homoeologous chromosomes is usually under polygenic control, with one locus having a greater influence than the others and frequent gene-dosage effects. *Brassica napus* has become a second model for deciphering how recombination and pairing between homoeologous chromosomes are genetically suppressed. The allotetraploid *B*. *napus* (AACC) shows complete diploid-like meiotic behaviour, with only bivalents at metaphase I (MI). In their dihaploids (AC), the amount of chromosome pairing at MI varies depending on the varieties the haploids originate from. This suggested the presence of a major locus, *PrBn*, which regulate cross over formation between homoeologous chromosomes [1, 6, 14]. The presence of regulator genes preventing homoeologous pairing in allopolyploids, where homoeologous genomes are in two doses, was also proposed in other plants [1, 14].

The meiotic behaviour of *Zea* species and artificial hybrids showed the affinity between genomes and revealed that *Zea* is a paleopolyploid complex, being maize and its allied species or teosintes “diploidized polyploids” with a basic number of five chromosomes (x = 5) [15–17]. It also was postulated that the genus is composed by allotetraploids species, with 2n = 20 chromosomes (maize and teosintes), and the alloautooctoploid species *Z*. *perennis* (2n = 40) [15–25]. These studies supported the polyploid condition of the genus *Zea* and the existence of two parental genomes, arbitrarily named A and B, which can be, in different species, homologous, homoeologous or non homologous. Moreover, the genomic formulae for all species of *Zea* were proposed, being A_m_A_m_B_m_B_m_ and A_x_A_x_B_x_B_x_ for maize and teosintes with 2n = 20 chromosomes, and A_p_A_p_A_p´_A_p´_B_p1_B_p1_B_p2_B_p2_ for *Z*. *perennis* (2n = 40) [15–17, 20, 22].

Colchicine applied at the onset of meiosis favors homoeologous chromosome pairing resulting in multivalent formation in bivalent-forming species. This spindle inhibitor suppresses the expression of pairing regulator genes and alters the premeiotic alignment of chromosomes [8, 26–29]. In wheat, Feldman and Levi [8] discussed the effect of different doses of *Ph1* on chromosome pairing, which can be phenocopied by premeiotic treatment with different concentration of colchicine. Cowan and Cande [30] demonstrated that colchicine induces changes in the nuclear architecture and inhibit the meiotic bouquet formation, which is defined by the aggregation of telomeres on a small region of the nuclear envelope. A failure of telomere clustering may result in unpaired chromosomes and, consequently, reduce both synapsis and recombination.

In *Zea*, Poggio and colleagues [17] and Naranjo and colleagues [16] showed, for the first time, the formation of up to five quadrivalents (IV) in maize (2n = 20), up to 10 IV in *Z*. *perennis* (2n = 40) and up to ten trivalents (III) in *Zea perennis* x *Z*. *diploperennis* hybrids (2n = 30), in material treated with colchicine 0.5mM. On this basis, these authors proposed that colchicine treatment favors homoeologous chromosome pairing by suppressing the expression of a maize locus equivalent to the *Ph1* of wheat, and that maize would be a segmental allopolyploid [17, 22]. Moreover, molecular analysis provided compelling evidence that maize is a segmental allopolyploid [31–37].

The aim of the present work is to analyze how cytological diploidization is achieved and/or genetically controlled in the paleolyploids species of the genus *Zea*. This process is a critical step for polyploid speciation and is fundamentally different in autopolyploid and allopolyploid species. Moreover, new insights into the cytological diploidization of the alloautopolyploid *Z*. *perennis*, where homologous and homoeologous genomes share the same nucleus, are discussed. It is also analyzed if the diploid-like meiotic behaviour of polyploids is the result of the divergence between homoeologous chromosomes and/or from the activity of pairing regulator homoeologous genes.

## Materials and methods

### Plant material

*Zea mays* ssp. *mays* (2n = 20), Argentine maize landrace Amarillo Chico (VAV 6451) from the Laboratorio Vavilov, Universidad de Buenos Aires (UBA). Dihaploid maize was obtained and legated by Ing. Agr. J. Correa [38]. *Zea perennis* (2n = 40) from Ciudad Guzman, Jalisco, Mexico and *Zea mays* ssp. *parviglumis* (2n = 20) from Balsas valley, Guerrero, Mexico were legated by Dr. Kato T. Y., Colegio de Postgraduados, México.

Interspecific crossings between *Z*. *perennis* 2n = 40 (female) and maize 2n = 20 (male), and between *Z*. *perennis* 2n = 40 (female) and *Zea mays* ssp. *parviglumis* 2n = 20 (male) were carried out in the greenhouse to obtain the F1 hybrid plants (2n = 30). About 20 plants of *Z*. *perennis* were hand-pollinated with a bulk of pollen from 5 male plants. The species and hybrids are cultivated in the greenhouse of the Facultad de Agronomía, UBA.

### Meiotic analysis

Colchicine treatment was made according to Jackson and Murray [27] with minor modifications [17]. The stems of *Z*. *perennis* and the hybrids, carrying the male tassel, were cut under a diluted solution of colchicine (Merck) (0.5 x 10–4 M, 0.5mM) and maintained therein for 12h (keeping the submerged portion of the stem in the dark). Before fixation, the stems were placed for 24h in tap water. In maize stems the same treatment were made at a high concentration of colchicine (1mM). Treated (T) and control-untreated (UT) materials were fixed in 3: 1 (absolute ethylic alcohol: acetic acid) solution. Anthers were squashed in 2% acetic haematoxylin. The meiotic configurations were studied at Diakinesis-Metaphase I. The results were analyzed in at least two individuals per treatment. A chi-square test (*X*^*2*^) was performed for the variable “number of multivalents” in T and UT individuals.

The fluorescent *in situ* hybridization technique (FISH), using 18S-rDNA and maize 180bp-knob sequences as probes, was carried out as described by Poggio and colleagues [18]. Slides were examined with a Carl Zeiss Axiophot epifluorescence microscope.

## Results

Stems of *Zea mays* ssp. *parviglumis* (2n = 20) were treated with colchicine 0.5mM and the meiotic behaviour showed 10II in all the studied cells in Prophase I.

**Fig 1:**
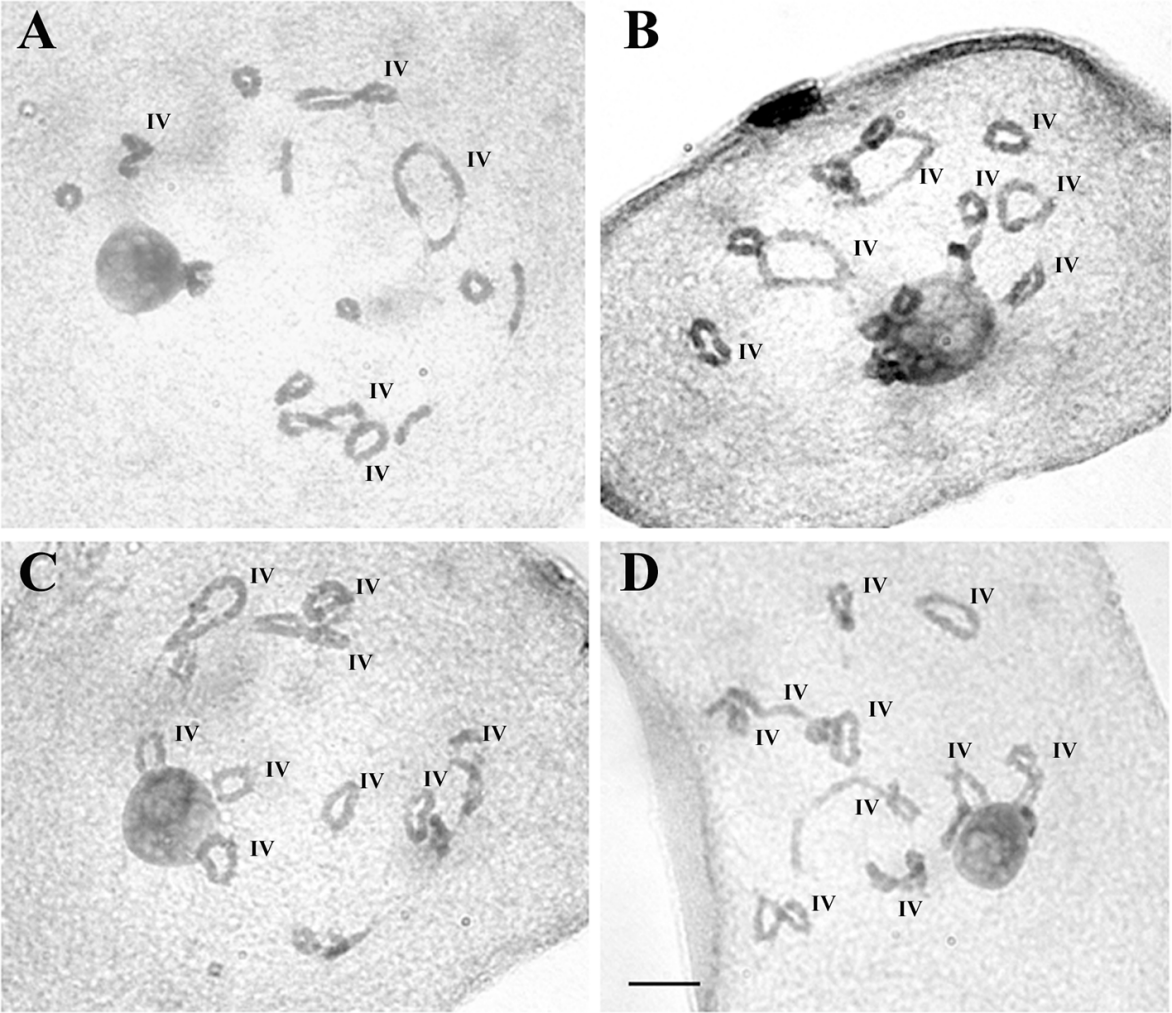
Meiotic configurations in *Z*. *perennis*. **A:** Untreated material, 5IV+10II. **B-D:** Treated material with colchicine 0.5mM. **B:** 7IV+6II. **C:** 9IV+2II. **D:** 10IV. Ref.: IV: quadrivalents. Bar: 10 μm.

Untreated (UT) *Zea perennis* (2n = 40) shows 5IV+10II in most of the analyzed cells, with a maximum of 6 IV (Table 1, Fig 1A). In the treated (T) material of *Z*. *perennis* an increase up to 10IV was found (Table 2, Fig 1B–1D). The difference in the number of multivalents in treated and untreated individuals of *Z*. *perennis* was highly significant (*X*^*2*^ = 117, p-value < 0.05).

**Table 1:** Meiotic configuration of untreated individuals of *Z*. *perennis*.

IV	II	N° cells	%
2	16	6	4.54
3	14	11	8.33
4	12	33	25
5	10	74	56.06
6	8	8	6.06
$\bar{X}$±SD 4±1.58	$\bar{X}$±SD 12±3.16	132	

IV: Quadrivalents. II: bivalents.

**Table 2:** Meiotic configuration of individuals of *Z*. *perennis* treated with colchicine 0.5mM.

IV	II	N° cells	%
3	14	3	1.81
4	12	19	11.51
5	10	34	20.60
6	8	42	25.45
7	6	42	25.45
8	4	16	9.69
9	2	6	3.63
10	-	3	1.81
$\bar{X}$±SD 6.5±2.45	$\bar{X}$±SD 7±4.9	165	

IV: Quadrivalents. II: bivalents.

**Fig 2:**
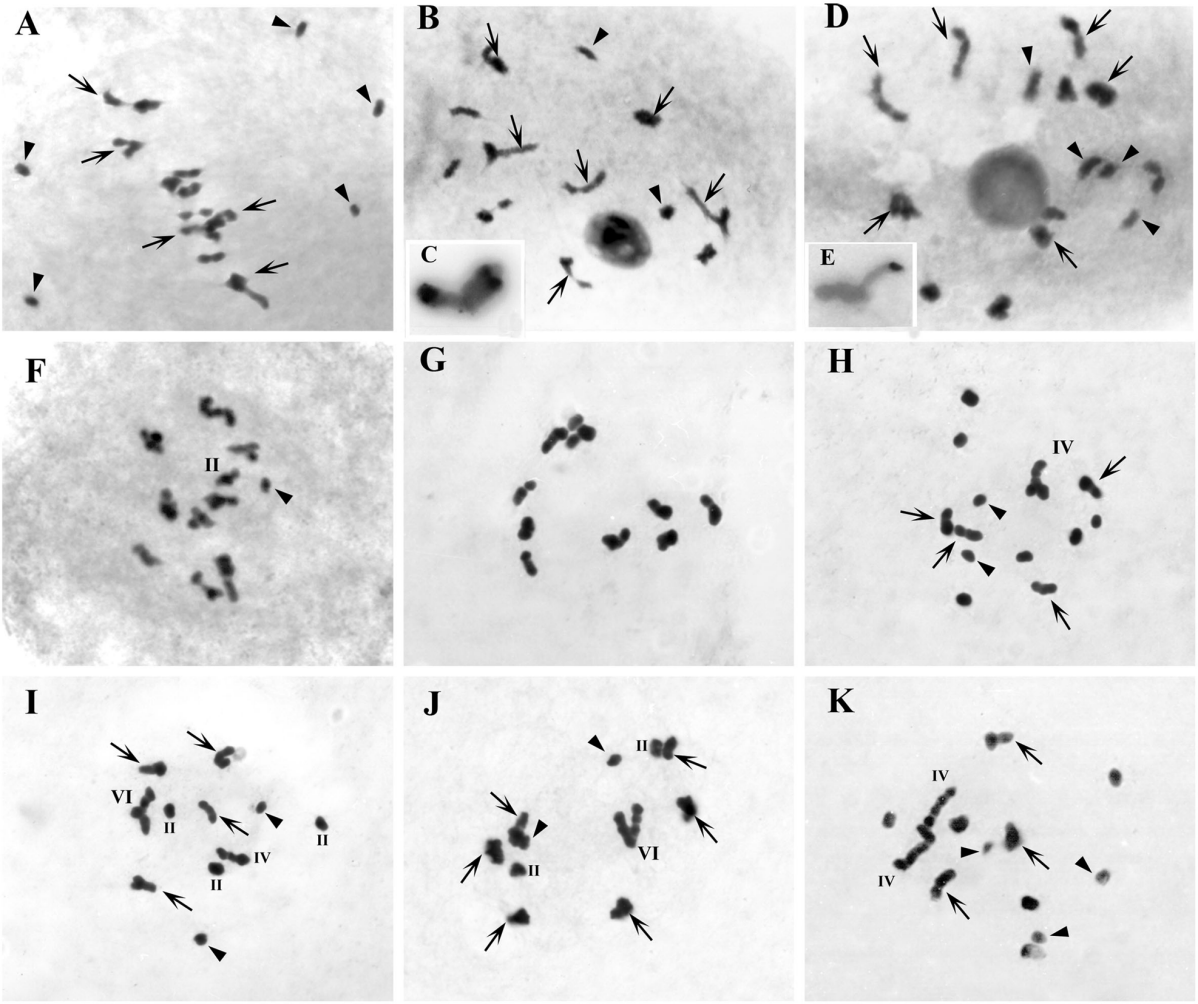
Meiotic configurations in 2n = 30 hybrids. **A, B, C, E:** Untreated *Z*. *perennis* x *Z*. *m*. ssp. *mays*: **A:** 5III+5II+5I, **B:** 6III+5II+2I, **C:** one trivalent shows three FISH signals of 18SrDNA maize probe, **E**: one trivalent shows one FISH signal of knob maize probe on the “handle”, corresponding to maize chromosome, of the trivalent “frying pan” configuration. **D:** Untreated *Z*. *perennis* x *Z*. *m*. ssp. *parviglumis*, 6III+4II+4I, one III associated to nucleoli. **F:** *Z*. *perennis* x *Z*. *m*. ssp. *parviglumis* treated with colchicine 0.5mM, 9III+1II+1I. **G-K:** *Z*. *perennis* x *Z*. *m*. ssp. *mays* treated with colchicine 0.5mM: **G:** 10III, **H:** 1IV+4III+6II+2I, **I:** 1VI+1IV+4III+3II+2I, **J:** 1VI+6III+2II+2I, **K:** 2IV+3III+5II+3I. Arrows indicate the trivalents. Arrowheads show the univalents. Ref.: I: univalents, II: bivalents, III: trivalents, IV: quadrivalents, VI: hexavalents. Bar: 10 μm.

The UT 2n = 30 hybrids *Z*. *perennis* x *Z*. *m*. ssp. *parviglumis* and *Z*. *perennis* x *Z*. *m*. ssp. *mays* show 5III+5II+5I as the most frequent chromosome association. A maximum of 8III and 7III was observed, respectively (Tables 3 and 4, Fig 2A, 2B and 2D).

**Table 3:** Meiotic configuration of untreated individuals of *Z*. *perennis* x *Z*. *m*. ssp. *parviglumis*.

III	II	I	N° cells	%
2	8	8	5	4.35
3	7	7	4	3.48
4	7	4	2	1.74
4	6	4	13	11.30
5	6	3	2	1.74
5	5	5	51	44.35
6	4	4	26	22.61
7	3	3	4	3.48
8	2	2	5	4.35
$\bar{X}$±SD 4.89±1.9	$\bar{X}$±SD 5.33±2	$\bar{X}$±SD 4.67±2	112	

III: trivalents. II: bivalents. I: univalents.

**Table 4:** Meiotic configuration of untreated individuals of *Z*. *perennis* x *Z*. *m*. ssp. *mays*.

III	II	I	N° cells	%
2	12	-	1	0.79
2	10	4	4	3.17
2	9	6	2	1.59
3	9	3	2	1.59
3	7	7	3	2.38
4	7	4	7	5.55
4	6	6	16	12.70
5	5	5	56	44.44
5	4	7	5	3.97
6	5	2	4	3.17
6	4	4	15	11.90
6	3	6	2	1.59
7	3	3	9	7.14
$\bar{X}$±SD 4.23±1.74	$\bar{X}$±SD 6.46±2.85	$\bar{X}$±SD 4.38±2.06	126	

III: trivalents. II: bivalents. I: univalents.

In T hybrid *Z*. *perennis* x *Z*. *m*. ssp. *parviglumis* the frequency of six or more trivalents increased from 30% in the UT material to 87% in the treated one (Table 5, Fig 2D and 2F).

**Table 5:** Meiotic configuration of treated individuals of *Z*. *perennis* x *Z*. *m*. ssp. *parviglumis*.

III	II	I	N° cells	%
3	7	7	1	0.77
4	6	6	3	2.32
5	5	5	13	10.07
6	4	4	17	13.17
7	3	3	28	21.7
8	2	2	35	27.13
9	1	1	23	17.82
10	-	-	9	6.97
$\bar{X}$±SD 6.5±2.45	$\bar{X}$±SD 3.5±2.45	$\bar{X}$±SD 3.5±2.45	129	

III: trivalents. II: bivalents. I: univalents.

The difference in the number of multivalents in T and UT individuals of *Z*. *perennis* x *Z*. *m*. ssp. *parviglumis* was highly significant (*X*^*2*^ = 113, p-value < 0.05).

The T 2n = 30 hybrids *Z*. *perennis* x *Z*. *m*. *ssp*. *mays* showed trivalents (III), quadrivalents (VI) and hexavalents (VI) (Table 6, Fig 2G–2K).

**Table 6:** Meiotic configuration of treated individuals of *Z*. *perennis* x *Z*. *m*. ssp. *mays*.

VI	IV	III	II	I	N° cells	%
-	-	4	6	6	1	0.65
-	-	5	5	5	7	4.54
-	-	6	4	4	16	10.39
-	-	7	3	3	17	11.04
-	-	8	2	2	21	13.64
-	-	9	1	1	24	15.58
-	-	10	-	-	11	7.14
-	1	4	5	4	3	1.95
-	1	5	4	3	4	2.60
-	1	6	3	2	4	2.60
-	1	7	2	1	6	3.90
-	1	8	1	-	3	1.30
-	2	3	5	3	1	0.65
-	2	6	2	-	1	0.65
1	-	8	-	-	1	0.65
1	-	4	4	4	5	3.25
1	-	5	3	3	8	5.19
1	-	6	2	2	6	3.90
1	-	7	1	1	2	1.30
1	1	3	4	3	1	0.649
1	1	5	2	1	1	0.649
1	1	4	3	2	1	0.649
1	2	3	3	1	1	0.649
1	2	4	2	-	1	0.649
2	-	2	4	4	1	0.649
2	-	3	3	3	1	0.649
2	-	4	2	2	3	1.95
2	1	2	3	2	1	0.649
2	2	2	2	-	1	0.649
3	1	2	1	-	1	0.649
$\bar{X}$±SD 0.76±0.86	$\bar{X}$±SD 0.66±0.76	$\bar{X}$±SD 5.06±2.21	$\bar{X}$±SD 2.73±2.51	$\bar{X}$±SD 2.06±2.66	154	

VI: hexavalents. IV: quadrivalents. III: trivalents. II: bivalents. I: univalents.

The difference in the number of multivalents in T and UT individuals of *Z*. *perennis* x *Z*. *m*. *ssp*. *mays* was highly significant (*X*^*2*^ = 162, p-value < 0.05).

FISH was carried out on meiotic metaphases of UT hybrids *Z*. *perennis* x *Z*. *m*. ssp. *mays* (2n = 30) using labeled 18S rDNA and knob-180bp maize sequences as probes. The 18S rDNA shows three fluorescence signals on a single trivalent (Fig 2C), indicating that the maize chromosome and the two *Z*. *perennis* chromosomes having the NOR sequences are homoeologous and are paired. The knobs are absent in *Z*. *perennis*, the knob-180bp maize sequence probe showed a strong fluorescence signal on only one of the chromosomes, on the “handle” of the “frying pan”-shaped trivalents (Fig 2E), showing that the two unlabelled chromosomes belong to the *Z*. *perennis* parent, while the labelled chromosome belongs to *Z*. *m*. ssp. *mays*.

**Fig 3:**
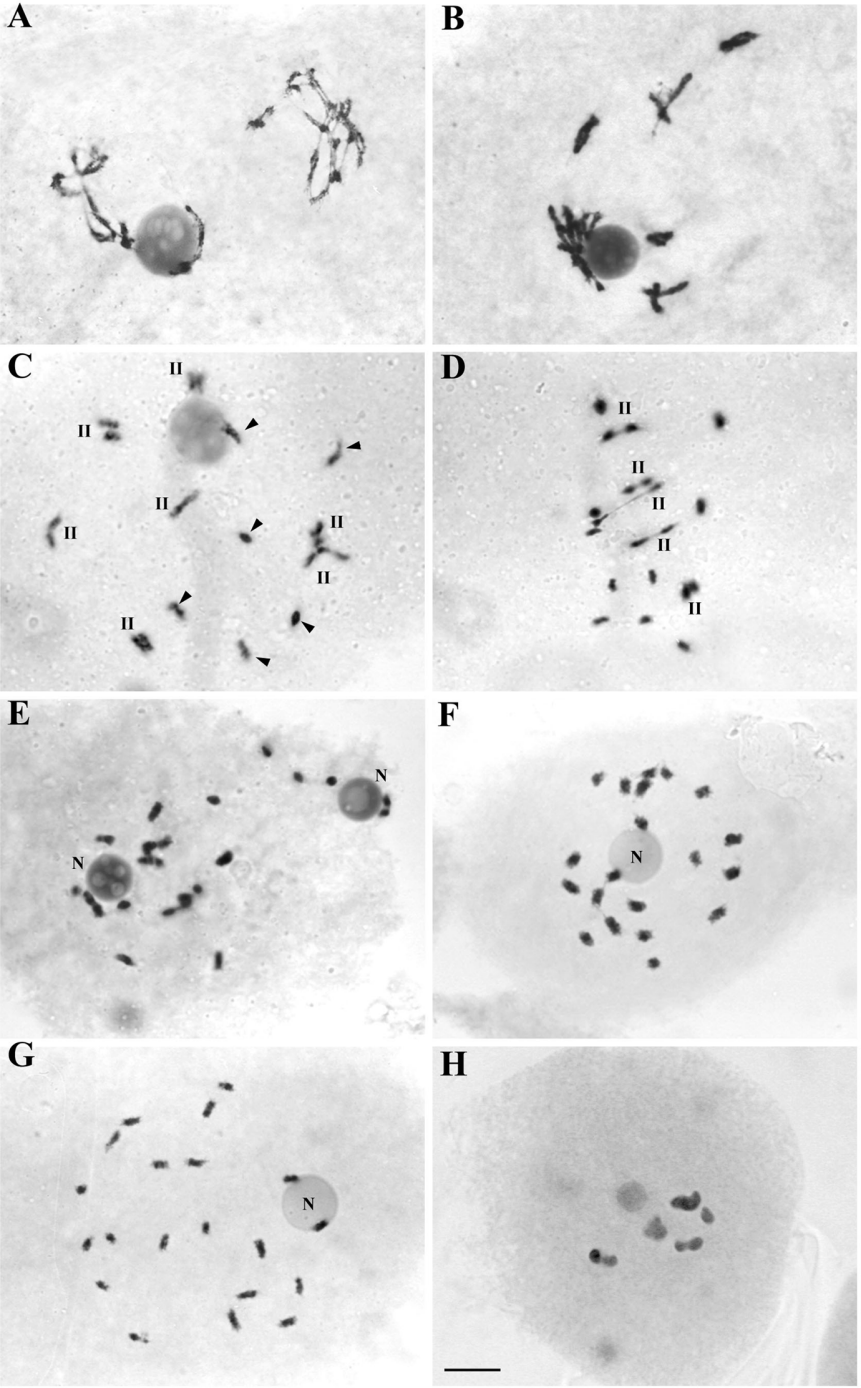
Meiotic chromosomes of *Zea mays* ssp. *mays*. **A-B, H:** Untreated material. **C-G:** Treated with colchicine (1mM). **A-B:** Diplotene-Diakinesis: asynchrony in two groups of five bivalents each. **C:** Diakinesis: 7 bivalents (II) with terminal chiasmata and 6 univalents (arrowhead). **D:** Metaphase I: 5II+10I. **E, F, G:** Total asynapsis and homologous separation; **E:** two nucleolus (N), one of them associated to 5 chromosomes, **F:** two chromosomes associated to the nucleolus (N), **G:** 20I, two of them associated to the nucleolus (N). **H:** Untreated dihaploid maize, 5II in Diakinesis. Bar: 10 μm.

In UT maize, two spatially separated groups of 5II each were observed in Prophase I (Fig 3A and 3B).

Differences in the meiotic configurations were found between two individual of maize treated with colchicine 1mM. One of the individuals showed cells with 7II+6I (15%) (Fig 2C), 6II+8I (20%) and 5II+10I (40%) (Fig 3D); also 15% of the 70 studied cells showed 2II+16I, 3II+14I and 4II+12I, while 10% presented total asynapsis with 20I (Fig 3F). The other individual showed most of the 42 studied cells (85%) with total asynapsis (20I) (Fig 3E and 3G); in some of these cells two nucleoli were observed and 5 chromosomes associated to one of them were separated from the rest (Fig 3E).

The meiosis of five UT dihaploid maizes showed 1–5 bivalents in the 14% of the 120 studied cells (Fig 3H), the rest presented 10I.

**Fig 4:**
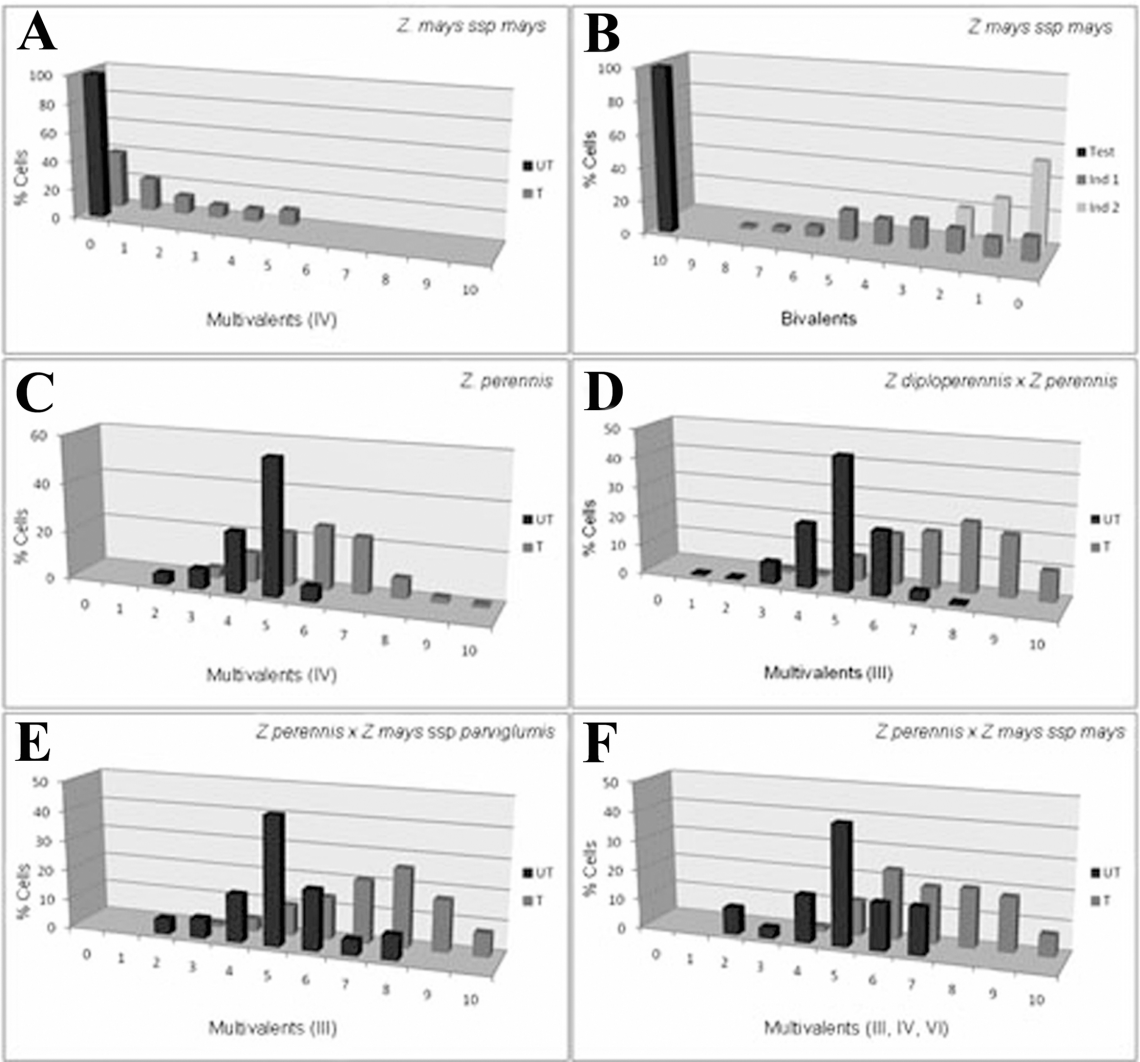
Frequency of multivalents and/or bivalents in treated (T) and untreated (UT) material. **A, C-F:** material treated with colchicine 0.5mM. **B:** material treated with colchicine 1mM. A: data from Poggio *et al*. (1990). D: data from Naranjo *et al*. (1994).

The histograms in Fig 4 represent the frequency of bivalents and multivalents in treated and untreated species and hybrids.

**Fig 5:**
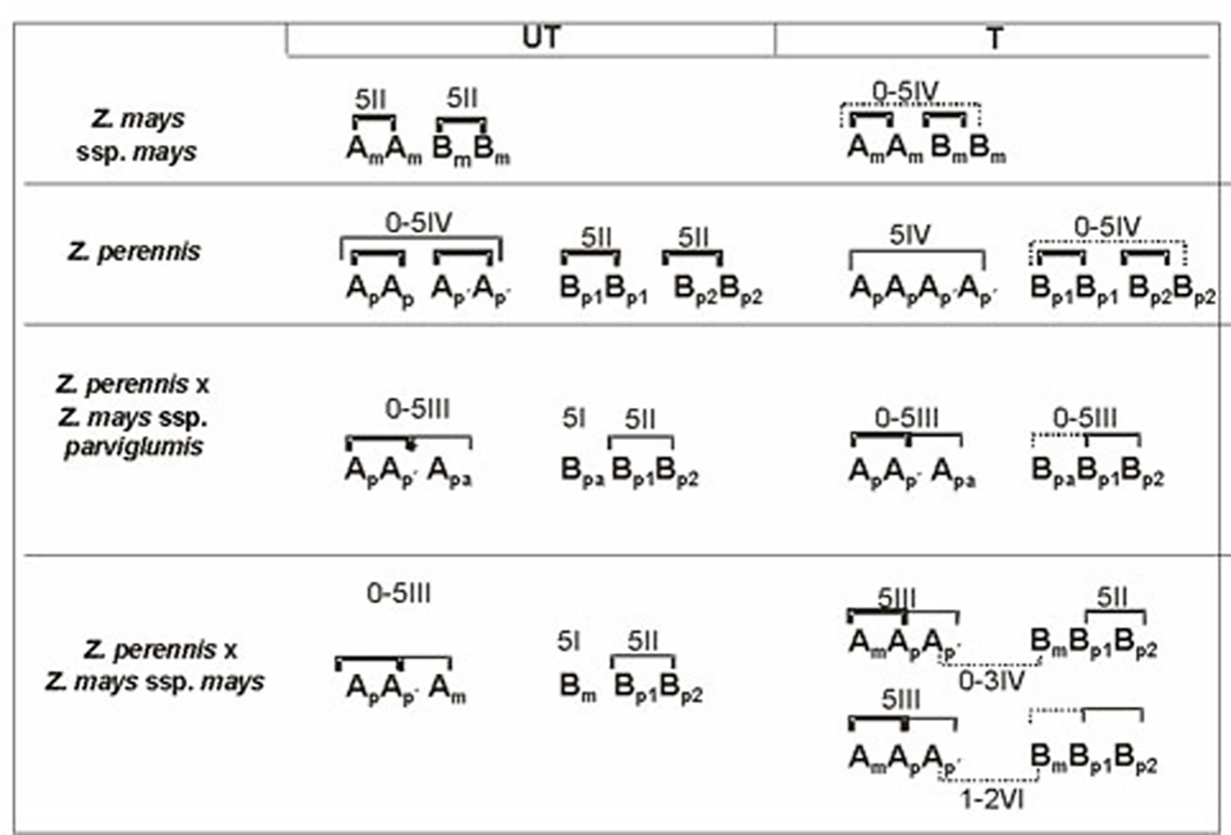
Genome formulae and the most frequent meiotic configurations for *Zea* species and hybrids (T) and (UT) with colchicine 0.5mM. Thick lines show the meiotic associations more frequently observed among homologous chromosomes, fine lines and dotted lines indicate meiotic associations among homoeologous chromosomes (dotted lines indicate less homoelogy than fine lines).

The genomic formulae and the parental genomic affinities revealed by the meiotic behaviour of species and hybrids, UT and T with colchicine 0.5mM, are summarized in Fig 5. In this figure the thick lines show the meiotic associations more frequently observed among homologous chromosomes, fine lines and dotted lines indicate meiotic associations among homoelogous chromosomes. Dotted lines show homoeology revealed by colchicine treatment.

## Discussion

### Pairing regulator genes in *Zea*: cytological evidence

In many plants colchicine applied at the onset of meiosis suppresses the effect of pairing regulator genes, resulting in formation of multivalents in bivalent-forming species [8]. In maize, treatment with colchicine 0,5mM showed the formation up to 5 quadrivalents, suggesting pairing between chromosomes from genomes homoeologous. These experiments led to postulate that maize possess a locus *Ph1*-like preventing pairing between homoeologous chromosomes from genomes A_m_ and B_m_, and whose expression, as in wheat, could be suppressed by colchicine treatments [16, 17]. These results confirm that colchicine could be a test of homology in organisms possessing pairing regulator genes. In fact, colchicine treatment allowed to demonstrated that in maize, a tetraploid that have evolved 11 million years ago, homoeologous pairing still occur, confirming that maize should be considered a segmental allotetraploid as was postulated by cytogenetical studies [15, 17, 22–24].

In the present work, maize treated with higher doses of colchicine (1mM) showed total asynapsis (20 univalents) and, in some cells, five chromosomes were notoriously spatially separated from the rest. These chromosomes correspond to the genome arbitrarily named A because it included the NOR chromosome [24]. The other five chromosomes, belonging also to A genome, remain together with the 10 chromosomes belonging to B genomes. This could be indicating that, in this experiment, colchicine 1mM treatment induced, in addition to asynapsis, spatial separation between chromosomes homologous as was reported in wheat by Feldman and Levy [8]. These authors found homologous separation in wheat treated with colchicine 1mM and assumed that each genome occupies a separate region in the nucleus, which in turn, is recognized by *Ph1* and affected by colchicine treatments. In another individual, the asynapsis occurred in only 10 chromosomes, being 5II+10I the most frequent configuration. These strongly suggest that asynapsis could have occurred in one genome (A or B). These assumption is based in previous work that propose that in species and hybrids of *Zea* the two spatially separated asynchronic groups of five bivalents each represent A and B ancestral genomes with x = 5 [15, 16, 20, 22, 24, 39, 40]. Therefore, meiotic behaviour of individuals treated with different doses of colchicine allowed to postulate that the pairing regulator gene proposed in maize (*PrZ*) affect the association of homoeologous by controlling entire chromosomal sets or genomes (A_m_ or B_m_) rather than individual chromosomes. The same effect was described in wheat by Feldman and Avivi [28].

Relevant cytological findings supporting the polyploid nature of maize was the existence of chromosome pairing in dihaploids [41, 42]. The formation up to five bivalents in dihaploids observed in the present work demonstrated the occurrence of homoeologous pairing between genomes A_m_ and B_m_. It is interesting to note that in maize (2n = 20) the formation up to 5IV in treated material indicated that pairing regulator gene proposed precluded homoeologous pairing between A_m_ and B_m_ when they are in two doses in the polyploids (A_m_A_m_B_m_B_m_). Then, the formation of bivalents in dihaploids (A_m_B_m_) indicates that this pairing regulator gene would be less effective when homoeologous genomes are in only one dose. On these bases, it could be concluded that the proposed *PrZ* gene/s resembles the *PrBn* gene reported in *Brassica napus*. In fact, the allotetraploid *B*. *napus* (AACC) showed diploid-like meiotic behaviour, with only bivalents at MI, and pairing in dihaploids (AC) was also reported [1, 6]. Incomplete dominance when homoeologous genomes are in only one dose was also reported in *Glandularia* [13].

Cytological diploidization process is fundamentally different in autopolyploid and allopolyploid species, because of their different chromosome composition. In autopolyploids cytological diploidization is viewed as an increased number of bivalents at MI in detriment of multivalents. The diploidized meiotic behavior of allopolyploids could be attributed to the activity of pairing regulator genes or to the divergence between homoeologous chromosomes [1, 6, 7].

The alloautooctoploid *Z*. *perennis* (2n = 40), with a genomic formulae A_p_A_p_A_p´_A_p´_B_p1_B_p1_B_p2_B_p2_, offers an exceptional opportunity to analyze, in the same nucleus, the diploidization of highly homologous (A_p_A_p_A_p´_A_p´_) and homoeologous (B_p1_B_p1_ and B_p2_B_p2_) genomes. The meiotic studies of UT individuals of *Z*. *perennis* revealed that 5IV+10II was the most frequent chromosome configuration, in agreement with data previously reported [15, 22, 24, 40]. Poggio and colleagues [17] postulated that, in UT *Z*. *perennis*, the IV are formed by pairing of homologous chromosomes from A genomes (A_p_A_p_A_p´_A_p´_), while the II are formed by pairing of genomes B _p1_ (B_p1_B_p1_) and genomes B_p2_ (B_p2_B_p2_), respectively (Fig 5).

*Z*. *perennis* treated with colchicine shows up to 10IV indicating homoeology between genomes A_p_ (A_p_A_p_A_p´_A_p´_) and between genomes B_p_ (B_p1_B_p1_B_p2_B_p2_), but any homology among them. The pairing between B_p1_ and B_p2_ was confirmed by GISH in the *Z*. *perennis* x maize hybrids by Gonzalez and colleagues [24]. This strongly suggests that in UT *Z*. *perennis* studied in the present work, homoeologous pairing between the genomes B_p1_ and B_p2_ is prevented by a pairing regulator gene. However, the presence in high frequency of 5IV in UT individuals suggest that this genes would not affect the A genomes. This could be explained if A genomes are highly homologous, and more divergence in these genomes is required for the suppression of the homoeologous pairing. These results would indicate that the *PrZ* gene would affect independently the genomes A and B in *Z*. *perennis*, being relevant the threshold of homology and the fidelity of pairing between genomes A (A_p_ and A_p´_) and between genomes B (B_p1_ and B_p2_). The meiotic analysis of both genomes leads to conclude that *Z*. *perennis* is a combination of an intervarietal autotetraploid (A genomes) combined with a segmental allopolyploid (B genomes) in a same nucleus.

When *Z*. *m*. ssp. *parviglumis* (2n = 20) was treated with colchicine 0.5mM, no IV were observed, being 10II the meiotic configuration observed in Profase I, as was reported in treated *Z*. *diploperennis* by Poggio and colleagues [17]. In these tetraploid species, colchicine do not affect the meiotic behaviour, unlike what was observed in maize and the B_p_ genomes of *Z*. *perennis*, which showed up to five and up to ten IV, respectively. This could be explained if genomes A_x_ and B_x_ of *Z*. *m*. ssp. *parviglumis* and *Z*. *diploperennis* are differentiated and do not have regions prone to pair. Then, the absence of IV in treated *Z*. *m*. ssp. *parviglumis* and *Z*. *diploperennis* could be due to a real lack of homoeology between genomes A and B. On this basis, *Z*. *m*. ssp. *parviglumis* and *Z*. *diploperennis* would be typical genomic allopolyploids while maize is a segmental allopolyploid (Fig 5).

The 2n = 30 hybrids *Z*. *perennis* x *Z*. *m*. ssp. *mays* and *Z*. *perennis* x *Z*. *m*. ssp. *parviglumis* studied in this work showed, at meiosis of UT individuals, 5III+5II+5I as the most frequent chromosome configuration at MI. GISH experiments enabled the recognition of the genomic source of each chromosome involved in the meiotic configuration of the hybrids *Z*. *perennis* x *Z*. *m*. ssp. *mays* [24]. In this way, it was demonstrate that trivalents are formed by two chromosomes of genomes Ap (A_p_A_p´_) of *Z*. *perennis* and one chromosome of genome A (A_m_) of 2n = 20 parental, bivalents are formed by autosyndetic pairing of genomes B_p1_ and B_p2_ of *Z*. *perennis*, and univalents correspond to the genome B_m_ of maize [24 and present work]. Similar results were obtained in the hybrids *Z*. *perennis* x *Z*. *luxurians* [20]. All these results confirmed that genomes B_p1_ and B_p2_, that do not pair in *Z*. *perennis*, are homoeologous and form bivalents in the hexaploid hybrids which possesses only one doses of A_x_, B_x_, B_p1_ and B_p2_. This indicate that the *PrZ*-pairing regulator gene proposed for maize and *Z*. *perennis* is less efficient in dihaploid condition, as it was reported in dihaploids of *Brassica* and diploid hybrids of *Glandularia* [1, 13, 14]. Thus, all these results lead to postulated that *PrZ* preclude homoeologous pairing when homoeologous genomes are in two doses in the polyploids (B_p1_B_p1_B_p2_B_p2_) but are inefficient when homoeologous genomes B_p1_ and B_p2_ are in one dose in the hybrids.

When the hexaploid hybrid *Z*. *perennis* x *Z*. *m*. ssp. *parviglumis* was treated with colchicine 0.5 mM, up to 10III were observed. The same result was obtained in the hybrids *Z*. *perennis* x *Z*. *diploperennis* [17] and other hexaploid hybrids [43]. The absence of IV or VI in treated *Z*. *perennis* x *Z*. *m*. ssp. *parviglumis* is congruent with the lack of IV in treated *Z*. *m*. ssp. *parviglumis*, and reinforces that the genomes A_pa_ and B_pa_ of *Z*. *parviglumis* are not homoeologous between them (Fig 5).

Hexaploid hybrids *Z*. *perennis* x *Z*. *m*. ssp. *mays* treated with colchicine presented VI, IV and III at MI, these configurations differs notoriously from those observed in all the treated hexaploid hybrids involving *Z*. *perennis* and teosintes as parentals, where only III were observed. This result it is not unexpected and is explained by the homoeology found between genomes A_m_ and B_m_ of maize (Fig 5). This homoeology was demonstrated by pairing in dihaploids (A_m_B_m_) and the formation of IV in treated material of maize (A_m_A_m_B_m_B_m_).

### Cytological diploidization by restriction of pairing and/or genetical divergence of homoeologous chromosomes

Although the presence of pairing regulator genes is common in hybrids and polyploids, the mechanisms involved in this control were focusing on *Ph1* locus of wheat. Apart from this system, little is known about the activity of genes that contribute to the cytological diploidization of allopolyploids. It was postulated that *Ph1* of wheat exerts its effect at premeiotic stages controlling the presynaptic alignment of chromosomes. Moreover, it was demonstrated that different doses of *Ph1* influences the distance between homologous and homoeologous chromosomes [8]. Besides, these authors demonstrated that colchicine phenocopies the effect of increased doses of *Ph1* in premeiotic cells and postulated that the resemblance between the effect of *Ph1* and colchicine lead to the assumption that microtubules are the subcellular target of *Ph1*.

Interestingly, Feldman and Levy [8] showed that, in hexaploid wheat, each ancestral genome occupies a separate region in the nucleus, which in turn, is recognized by *Ph1*. In polyploid species and hybrids of *Glandularia*, pairing regulator genes affecting the distance between nuclear site attachments of its two parental genomes was also proposed [13]. The studies carried out so far in different plants seem to indicate that different doses of pairing regulator genes affect the distance between nuclear site attachments of ancestral or parental homoeologous genomes of polyploid species [8]. Moreover, there are many evidences indicating that *Ph*–like genes are also evolved in important meiotic mechanisms related with pairing and recombination [1, 6].

In *Zea* it could be postulated that the *PrZ* would affect the distance between the nuclear site attachment of its two ancestral genomes, A and B, restricting the pairing between their chromosomes. Now it is known that the spatial separation of homoeologous, parental or ancestral genomes in hybrids and polyploids species is a very common phenomenon [44, 45]. Moreover, it is interesting to point out that the spatial separation of relict homoeologous genomes of *Zea*, could restricts pairing between their chromosomes [22].

In the tetraploids teosintes *Z*. *diploperennis* and *Z*. *m*. ssp. *parviglumis* colchicine treatments do not reveal any homeology between genomes A_x_ and B_x_. These results lead to the conclusion that, in these species, the diploidized meiotic behaviour could be achieved by the divergence of homoeologous chromosomes belonging to genomes A and B. On these bases, *Z*. *diploperennis* and *Z*. *m*. ssp. *parviglumis* could be considered genomic allopolyploid.

The divergence between homoeologous genomes could be explained by the process of genetic diploidization, phenomena that occurs when genomes of different species are combined together within a single nucleus, such as in hybridization and polyploidy. This revolutionary process, where the genetic redundancy is erased, triggers gene silencing, gene elimination and/or transposon activation via genetic and epigenetic alterations [2, 3, 5, 46].

The cytological diploidization by restriction of pairing between homoeologous chromosomes or by genetical divergence of the homoeologous chromosomes are independent but complementary systems and could be acting jointly in the same nucleus. The effect of *PrZ* on the genomes A and B could be different depending fundamentally on the threshold of homology within them.

In *Z*. *perennis* both processes of cytological diplodization, divergence and pairing regulator genes, are complementary. In this alloautopolyploid a genetic system precluding pairing between genomes B was described in the present work. Besides, there are evidences of genetic diploidization such as genome downsizing and lost of repetitive sequences [40].

Although *Pr* genes are not a prerequisite for regular disomic inheritance, they would be selected because enhance polyploid fitness [1, 4, 7]. Thus, pairing regulator genes could promote and speed the time required for correct chromosomal segregation becomes established. Once regular disomic inheritance is established by *Pr* genes, divergence between homoeologous chromosomes will become exacerbated and *Pr* genes could be retained or lost. Even though these genes could be lost in the process of divergence, it is hardly possible to consider that pairing regulator genes were retained in *Zea* species that have undergone divergence between homoeologous genomes such as *Z*. *diploperennis* and *Z*. *m*. ssp. *parviglumis*. This presumption is based on the fact that the molecular and cell biological characterization of *Ph1*-wheat suggested that these genes are a master coordinator gene that is involved in conserved meiotic processes across the Kingdoms [6].

This work provide new insight into the processes and genetic control of diploid-like meiotic behaviour in ancient polyploids from genus *Zea* and lead to discuss how correct chromosome segregation could be ensured in autopolyploid, allopolyploid and alloautopolyploid species. The cytological diplodization is an essential process for understand polyploid speciation since the evolution of polyploid species is closely linked to the nature of meiotic stabilization.
